# Clinical and genomic analysis of baseline and acquired *MET* fusions with an intact kinase domain in lung cancer patients

**DOI:** 10.1016/j.gendis.2023.02.046

**Published:** 2023-04-05

**Authors:** Bo Jin, Yutong Ma, Qian Wu, Qiuxiang Ou, Yang Shao, Shun Xu

**Affiliations:** a Department of Medical Oncology, The First Hospital of China Medical University, Shenyang, Liaoning 110001, China; b Geneseeq Research Institute, Nanjing Geneseeq Technology Inc., Nanjing, Jiangsu 210000, China; c School of Public Health, Nanjing Medical University, Nanjing, Jiangsu 211166, China; d Department of Thoracic Surgery, The First Hospital of China Medical University, Shenyang, Liaoning 110001, China

*MET* gene alterations in lung cancer patients mainly include exon 14 skipping and gene amplification, which are the key therapeutic targets and drive resistance to tyrosine kinase inhibitors (TKIs).^1^ However, the structural variants of *MET*, such as *MET* fusions, are much rarer (0.26%), as reported in a Chinese non-small cell lung cancer (NSCLC) cohort.^2^ Several recurrent *MET* fusions, such as *KIF5B-MET* and *HLA-DRB1-MET*, were reported as oncogenic drivers and showed favorable responses to crizotinib.^3^^,^^4^ In addition, *MET* fusions have been found to mediate resistance to EGFR-TKIs. With the application of comprehensive genomic analyses in clinical samples, an increasing number of *MET* fusion partners have been identified; however, the clinical and molecular characteristics of patients harboring such *MET* fusions remain to be investigated in large cohorts.

In this retrospective study, a total of 47 MET fusions with an intact kinase domain (KD) were detected in 44 lung cancer patients (Fig. 1A and Table S1, 2) whose baseline and/or post-treatment samples underwent capture-based hybrid targeted next-generation sequencing (NGS) between April 2016 and December 2021. The breakpoints within the *MET* gene spanned almost the complete coding sequence (CDS), and 20 breakpoints were in exon 15, adjacent to the tyrosine kinase domain. Nearly half (21/47, 44.7%) of the partner breakpoints were in intergenic regions (IGRs), which were referred to as IGR*-MET* (Fig. 1B). Canonical *MET* fusions with known gene partners accounted for the remaining 55.3%, with the recurrent fusion partner genes including *HLA-DRB1* (*n* = 4), *ST7* (*n* = 4), *CAPZA2* (*n* = 3), *CD47* (*n* = 2), and *HLA-DRB5* (*n* = 2).

**Figure 1:**
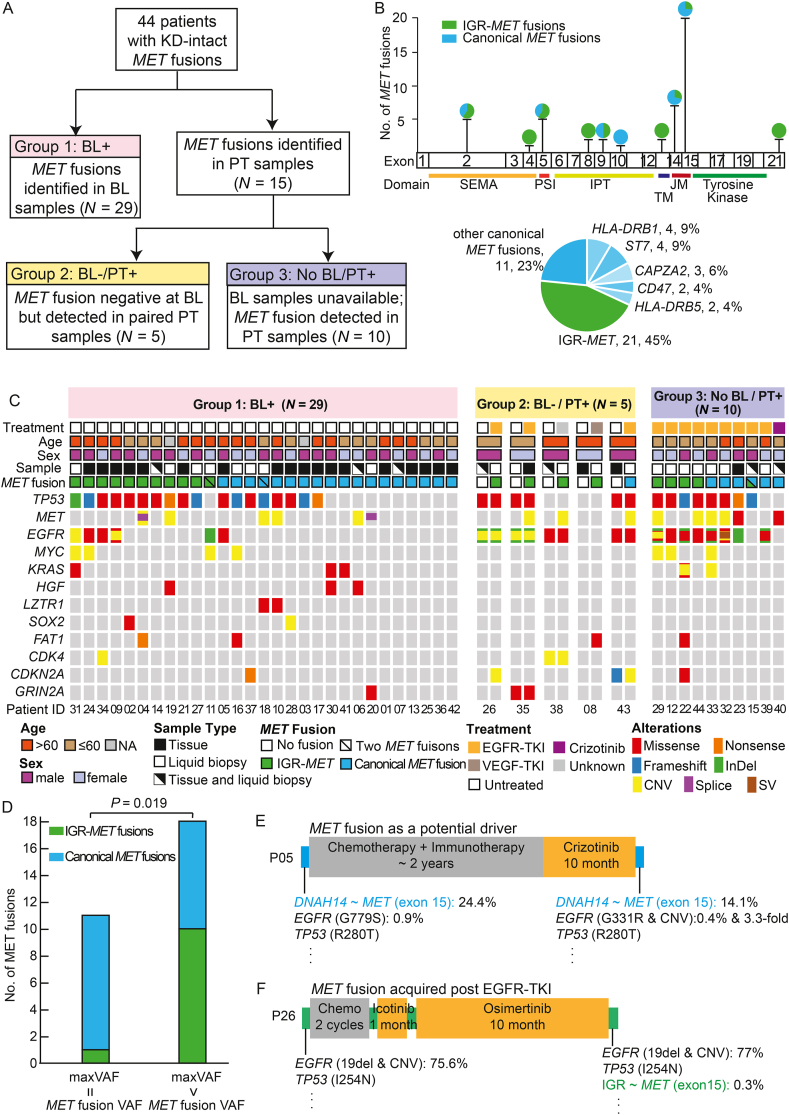
Molecular features of kinase domain-intact *MET* fusions. **(A)** The identified *MET* fusions were classified into three subgroups based on the sequenced sample timepoint (BL or PT) and baseline sample availability. **(B)** The number of *MET* fusions at each breakpoint is shown by the lollipop chart demonstrating the distribution of IGR-*MET* and canonical *MET* fusions. The distribution of recurrent fusion partners, IGR-*MET*, and other non-recurrent canonical fusions is shown in the pie chart. **(C)** The concurrent alterations in patients with *MET* fusions detected in BL samples (left panel), patients who acquired *MET* fusions with paired BL and PT samples (middle panel), and *MET*-fusion-positive patients without BL samples (right panel) are shown by the oncoprint plots. **(D)** The number of baseline IGR-*MET* and canonical *MET* fusions are shown in the bar plot, and these fusions are subgrouped based on the relationship between maxVAF and *MET* fusion VAF. **(E, F)** Treatment information (drug and duration) for two patients with *MET* fusions in BL and/or PT samples are shown with their representative mutations. BL, baseline; CNV, copy number variant; IGR, intergenic region; InDel, inframe insertion and deletion; IPT, immunoglobulin-like plexin transcription factor domain; JM, juxtamembrane domain; NA, not available; PSI, plexin, semaphorin, and integrin domain; PT, post-treatment; SEMA, semaphoring domain; SV, structure variant; TM, transmembrane domain; VAF, variant allele frequency.

*MET* fusions were detected in the baseline samples of 29 patients (Group 1), a small proportion of which were accompanied by *MET* amplification (17.2%, 5/29) and *MET* exon 14 skipping (6.9%, 2/29; Fig. 1C). The most frequent concurrent mutation was *TP53* (62.1%), which was strongly associated with IGR-*MET* (90.9% *vs*. 47.1%, *P* = 0.041). Similarly, *EGFR* mutations were significantly enriched in IGR-*MET* samples (45.5% *vs*. 5.6%, *P* = 0.018).

Notably, 19 patients had multiple baseline samples, including tissue, plasma, cerebrospinal fluid, and pleural effusion (Fig. S1A). However, the concordance of *MET* fusion detection between baseline plasma and tissue samples was only 33.3% (5/15), while that of plasma and other liquid biopsies exhibited a much higher consistency (4/5, 80%). The variant allele frequencies (VAFs) of *MET* fusions in 11 baseline samples were the highest among all detected alterations (maxVAF = *MET* fusion VAF), suggesting a potential oncogenic role for those *MET* fusions (Fig. 1D). Interestingly, the potential oncogenic *MET* fusions were significantly enriched as canonical *MET* fusions (10/11, 90.9%) compared to the passenger-like subgroup (8/18, 44.4%; *P* = 0.019). For example, P05 with a baseline *DNAH1*4-MET fusion as a potential driver benefited from crizotinib treatment for ten months (Fig. 1E). Mutational signature analysis of baseline samples with *MET* fusions revealed that dMMR (deficient DNA mismatch repair) was the most common mutational signature (Fig. S1B). Comparing IGR-*MET* and canonical *MET* fusion subgroups, significant differences were observed in mutational signatures related to age, ultraviolet exposure, and temozolomide treatment. Furthermore, tumor mutational burden (Fig. S1C) and chromosome instability (Fig. S1D) were also analyzed but were not affected by fusion partner (IGR and canonical) or oncogene concurrence.

In addition to the *MET* fusions detected in baseline samples, 15 patients (Group 2 and Group 3) harbored *MET* fusions in their post-treatment samples; however, five of them had paired baseline samples that lacked *MET* fusions (Fig. 1A). A total of 60% (3/5) of patients in Group 2 acquired *MET* fusions following *EGFR* TKI treatment (Fig. 1C). As shown in Figure 1F, P26 acquired an IGR-*MET* fusion following icotinib and osimertinib treatment, without other known resistance mutations, thus, suggesting a possible mechanism of EGFR-TKI resistance.

The first *MET* fusion in a lung cancer patient was discovered in 2017 as an *HLA-DRB1-MET* fusion. The fusion was identified using an anchored multiplex PCR (AMP)-based NGS assay in an early-stage lung adenocarcinoma patient who exhibited a robust response to crizotinib.^4^ Later, the oncogenic role of *MET* fusions was uncovered by functional *in vitro* experiments.^3^ In our cohort, P05 also benefited from crizotinib and harbored a *DNAH1*4-MET fusion, which might be the oncogenic driver. As for passenger-like *MET* fusions (*e.g.*, P09), they may not affect the responses to EGFR-TKIs for patients with actionable *EGFR* mutations.

Zhuo et al identified only 15 patients with *MET* KD rearrangements from 5965 Chinese NSCLC cases (0.25%), two-thirds (10/15) of which were canonical *MET* fusions.^2^ Similar to our findings, both baseline and acquired *MET* fusions were identified in their study, and one patient acquired a *TES-MET* fusion after 13 months of treatment with icotinib. In the current study, we identified five acquired *MET* fusions following treatment with EGFR- or VEGR-TKIs, which may represent potential resistance mechanisms, especially in patients without canonical resistance mutations. However, due to the low incidence of *MET* fusions, we could not validate our findings in external public datasets with large cohort sizes. Additionally, this was a single-center study with potential regional bias and the restricted cohort size may have also weakened the power of our statistical analyses.

Currently, *MET* fusions are not considered biomarkers for the administration of TKIs as *MET* exon 14 skipping. Cheng et al investigated the genomic and clinical characteristics of *MET* exon 14 skipping in a large Chinese cohort and identified 175 lung cancer patients with such alterations.^5^ The most frequently altered genes accompanied by *MET* exon 14 skipping were *TP53* (43%) and *EGFR* (17%). Similar findings were also observed in our study. Notably, however, only one active clinical trial (NCT04739358) investigating the efficacy of tepotinib in MET-driven NSCLC patients with central nervous system metastasis included NGS-detected *MET* fusions as an eligibility criterion. Thus, the clinical impacts of *MET* fusions remain to be investigated in prospective studies or clinical trials.

Our study investigated the largest dataset of *MET* fusions in lung cancer patients to date, and not only systematically investigated fusion partners, subtypes, and breakpoint preference, but also characterized concurrent mutation profiles, mutational signatures, and the tumor mutational burden of different *MET* fusion subtypes such as IGR-*MET* versus canonical fusions. Thus, the findings of this study provide valuable information for understanding the oncogenic or resistance mechanisms of *MET* fusions.

In conclusion, we comprehensively investigated the clinical and molecular characteristics of *MET* fusions in the largest lung cancer cohort to date. Both baseline and acquired *MET* fusions were identified, which might serve as oncogenic and resistance mechanisms against EGFR-TKIs, respectively. In addition, the NGS of multiple sample types could promote the use of personalized treatments by providing comprehensive molecular portraits.

## Ethics declaration

This study was approved by the Ethics Committee for the First Hospital of China Medical University (No. 20180551230). Written informed consent was obtained from each patient prior to sample collection and publication.

## Author contributions

BJ: conceptualization, methodology, formal analysis, data curation, writing-original draft. YM: formal analysis, visualization, writing-original draft. QW: visualization, investigation, writing-original draft. QO: validation, writing-review & editing. YS: validation, writing-review & editing. SX: supervision, project administration, writing-review & editing.

## Data availability

All data generated or analyzed during this study are included in this published article and its supplementary information files.

## Conflict of interests

Yutong Ma, Qian Wu, Qiuxiang Ou, and Yang Shao are employees of Nanjing Geneseeq Technology Inc., Nanjing, Jiangsu, China. The other authors declare no competing financial interests.
