# Supplementary material for: Clinical and genomic analysis of baseline and acquired MET fusions with an intact kinase domain in lung cancer patients
Source: Genes Dis. 2023 Apr 5;11(1):76–9. doi: 10.1016/j.gendis.2023.02.046 (PMC10425795; doi:10.1016/j.gendis.2023.02.046)
Supplement: Multimedia component 1 [file mmc1.docx]

**Supplementary File**

**Supplementary Methods**

**Supplementary Table S1. Clinical characteristics of patients with KD-intact *MET* fusions**

**Supplementary Table S2. Information of the 47 *MET* fusions detected in 44 patients**

**Supplementary Figure S1. Concordance of *MET* fusion detection in multiple sample types and subgroup analysis of TMB and CIS in baseline samples**

**Supplementary Methods**

*Next-generation sequencing (NGS)*

The NGS tests were conducted by a Clinical Laboratory Improvement Amendments-certified, College of American Pathologists-accredited laboratory (Nanjing Geneseeq Technology Inc., Jiangsu, China) as previously described.[1] In brief, genomic DNA was purified from FFPE samples using the QIAamp DNA FFPE Tissue Kit (Qiagen) and circulating tumor DNA (ctDNA) from liquid biopsies were extracted with the Circulating Nucleic Acid Kit (Qiagen) following the manufacturer’s protocol. The DNA quantification was performed using the dsDNA HS Assay Kit on a Qubit Fluorometer (Life Technologies). Sequencing libraries were prepared using the KAPA Hyper Prep Kit (KAPA Biosystems). Indexed DNA libraries were pooled together for probe-based hybridization capture of the targeted gene regions.

Sequencing was performed using the Illumina HiSeq4000 platform, followed by data analysis as previously described.[2] In brief, low-quality (quality <15) or N bases were removed and then the sequencing reads were mapped to the human reference genome, hg19, using the Burrows-Wheeler Aligner (https://github.com/lh3/bwa/tree/master/bwakit). PCR duplicates were removed by Picard (available at: https://broadinstitute.github.io/picard/). The Genome Analysis Toolkit (GATK) (https://software.broadinstitute.org/gatk/) was used to perform local realignments around indels and base quality reassurance. Somatic single-nucleotide polymorphisms (SNPs) and indels were analyzed by VarScan2 and Mutect2.[3] Common SNPs were excluded if they were present in >1% population frequency in the 1000 Genomes Project or the Exome Aggregation Consortium (ExAC) 65,000 exomes database. Gene fusions were identified by FACTERA,[4] and the allele frequency cutoff of *MET* fusion was 0.1%.

*Statistical analysis and survival analysis*

Data were analyzed using R 3.6.3. Categorical variables between groups were compared using χ2 or Fisher’s exact test. Non-parametric comparisons of TMB and mutational signature were performed using the Mann-Whitney test.

**Reference**

1. Shu, Y., et al., *Circulating Tumor DNA Mutation Profiling by Targeted Next Generation Sequencing Provides Guidance for Personalized Treatments in Multiple Cancer Types.* Sci Rep, 2017. **7**(1): p. 583.

2. Yang, Z., et al., *Investigating Novel Resistance Mechanisms to Third-Generation EGFR Tyrosine Kinase Inhibitor Osimertinib in Non-Small Cell Lung Cancer Patients.* Clin Cancer Res, 2018. **24**(13): p. 3097-3107.

3. Koboldt, D.C., D.E. Larson, and R.K. Wilson, *Using VarScan 2 for Germline Variant Calling and Somatic Mutation Detection.* Curr Protoc Bioinformatics, 2013. **44**: p. 15 4 1-17.

4. Newman, A.M., et al., *FACTERA: a practical method for the discovery of genomic rearrangements at breakpoint resolution.* Bioinformatics, 2014. **30**(23): p. 3390-3.

**Supplementary Table S1. Clinical characteristics of patients with KD-intact *MET* fusions**

|  | All patients | Group 1: BL+ | Group 2: BL-/PT+ | Group 3: No BL/PT+ |
| --- | --- | --- | --- | --- |
| No. of patients | 44 | 29 | 5 | 10 |
| Age: N (%) |  |  |  |  |
| ≤ 60 | 20 (45.5) | 11 (37.9) | 2 (40.0) | 7 (70.0) |
| > 60 | 22 (50.0) | 16 (55.2) | 3 (60.0) | 3 (30.0) |
| Unknown | 2 (4.5) | 2 (6.9) | - | - |
| Sex: N (%) |  |  |  |  |
| Female | 20 (45.5) | 12 (41.4) | 2 (40.0) | 6 (60.0) |
| Male | 24 (54.5) | 17 (58.6) | 3 (60.0) | 4 (40.0) |
| Histology: N (%) |  |  |  |  |
| ADC | 33 (75.0) | 21 (72.4) | 3 (60.0) | 9 (90.0) |
| ASC | 1 (2.3) | - | - | 1 (10.0) |
| Unknown | 10 (22.7) | 8 (27.6) | 2 (40.0) | - |
| Stage: N (%) |  |  |  |  |
| IV | 22 (50.0) | 11 (37.9) | 3 (60.0) | 8 (80.0) |
| Unknown | 22 (50.0) | 18 (62.1) | 2 (40.0) | 2 (20.0) |
| Treatments: N (%) |  |  |  |  |
| EGFR TKIs | 13 (29.5) | 1 (3.4) | 3 (60.0) | 9 (90.0) |
| Crizotinib | 3 (6.8) | 2 (6.9) | 0 (0.0) | 1 (10.0) |
| VEGF TKI | 1 (2.3) | - | 1 (20.0) | - |
| Untreated/Unknown | 27 (61.4) | 26 (89.7) | 1 (20.0) | - |

Note: NA, not available; ADC, adenocarcinoma; ASC, adenosquamous carcinoma; BL, baseline; PT, post-treatment; TKI, tyrosine kinase inhinitor.

**Supplementary Table S2. Information of the 47 *MET* fusions detected in 44 patients**

| **ID** | **MET fusions** | **Subgroup** | **Sample type** | **Allele Frequency** |
| --- | --- | --- | --- | --- |
| P1 | HLA-DRB5:exon4~MET:exon15 | BL+ | BL tissue | 5.2% |
| P10 | ST7:exon1~MET:exon15 | BL+ | BL tissue | 9.1% |
| P11 | IGR~MET:exon15 | BL+ | BL hydrothorax; BL plasma | 6.4%; 0.9% |
| P11 | MET:exon21~IGR | BL+ | BL hydrothorax; BL plasma | 26.4%; 4.4% |
| P13 | CAPZA2:exon1~MET:exon5 | BL+ | BL tissue | 3.0% |
| P14 | IGR~MET:exon14 | BL+ | BL tissue; BL plasma | 23.8%; 6.2% |
| P16 | GPRC5C:exon2~MET:exon15 | BL+ | BL hydropericardium | 1.2% |
| P17 | HLA-DRB1:exon5~MET:exon15 | BL+ | BL tissue | 37.5% |
| P18 | ST7:exon9~MET:exon15 | BL+ | BL plasma | 0.1% |
| P18 | CAPZA2:exon5~MET:exon15 | BL+ | BL plasma | 0.1% |
| P19 | IGR~MET:exon2 | BL+ | BL plasma | 0.2% |
| P2 | IGR~MET:exon8 | BL+ | BL tissue | 4.6% |
| P20 | CAPZA2:exon10~MET:exon2 | BL+ | BL plasma | 1.6% |
| P21 | IGR~MET:exon15 | BL+ | BL tissue | 4.2% |
| P24 | MET:exon21~IGR | BL+ | BL tissue | 6.3% |
| P25 | KIF5B:exon16~MET:exon14 | BL+ | BL tissue | 2.4% |
| P27 | IGR~MET:exon13 | BL+ | BL plasma | 0.2% |
| P28 | CD47:exon5~MET:exon15 | BL+ | BL tissue | 16.7% |
| P3 | CD74:exon5~MET:exon15 | BL+ | BL tissue | 2.5% |
| P30 | SEMA3D:exon7~MET:exon9 | BL+ | BL tissue | 11.6% |
| P31 | IGR~MET:exon2 | BL+ | BL plasma | 1.0% |
| P34 | IGR~MET:exon13 | BL+ | BL tissue | 1.6% |
| P36 | ST7:exon1~MET:exon5 | BL+ | BL tissue | 12.5% |
| P37 | HLA-DRB1:exon5~MET:exon14 | BL+ | BL hydrothorax; BL plasma | 1.5%; 1.8% |
| P4 | IGR~MET:exon8 | BL+ | BL tissue | 3.9% |
| P41 | GRM3:exon2~MET:exon2 | BL+ | BL tissue | 1.6% |
| P42 | EPHB4:exon10~MET:exon15 | BL+ | BL tissue | 8.6% |
| P5 | DNAH14:exon10~MET:exon15 | BL+ | BL tissue; PT plasma | 24.4%; 14.1% |
| P6 | HLA-DRB1:exon4~MET:exon15 | BL+ | BL tissue; BL plasma | 57.5%; 0.1% |
| P7 | HLA-DRB5:exon4~MET:exon14 | BL+ | BL tissue; BL plasma | 7.2%; 3.3% |
| P9 | IGR~MET:exon5 | BL+ | BL tissue | 4.3% |
| P26 | IGR~MET:exon15 | BL-/PT+ | PT plasma | 0.3% |
| P35 | IGR~MET:exon4 | BL-/PT+ | PT tissue (metastic) | 1.0% |
| P38 | IGR~MET:exon2 | BL-/PT+ | PT hydrothorax | 1.3% |
| P43 | WMT2:exon3~MET:exon15 | BL-/PT+ | PT hydrothorax | 5.3% |
| P8 | IGR~MET:exon15 | BL-/PT+ | PT plasma; PT hydrothorax | 0.9%; 0.5% |
| P12 | IGR~MET:exon5 | No BL/PT+ | PT plasma | 0.6% |
| P15 | CD47:exon3~MET:exon15 | No BL/PT+ | PT tissue (metastic); PT plasma-1; PT plasma-2 | 2.6%; 0.3%; 26.6% |
| P15 | IGR~MET:exon15 | No BL/PT+ | PT tissue (metastic); PT plasma | 1.2%; 3.9% |
| P22 | IGR~MET:exon9 | No BL/PT+ | PT plasma | 2.0% |
| P23 | ST7:exon2~MET:exon15 | No BL/PT+ | PT tissue | 1.3% |
| P29 | IGR~MET:exon14 | No BL/PT+ | PT plasma | 0.1% |
| P32 | CTTNBP2:exon4~MET:exon15 | No BL/PT+ | PT plasma | 0.4% |
| P33 | LSMEM1:exon5'UTR~MET:exon10 | No BL/PT+ | PT plasma | 2.6% |
| P39 | FOXP2:exon1~MET:exon14 | No BL/PT+ | PT plasma-1; PT plasma-2 | 1%; 0.1% |
| P40 | HLA-DRB1:exon5~MET:exon14 | No BL/PT+ | PT tissue; PT plasma-1; PT plasma-2 | 15.8%; 0.8%; 7.8% |
| P44 | IGR~MET:exon5 | No BL/PT+ | PT plasma | 0.3% |


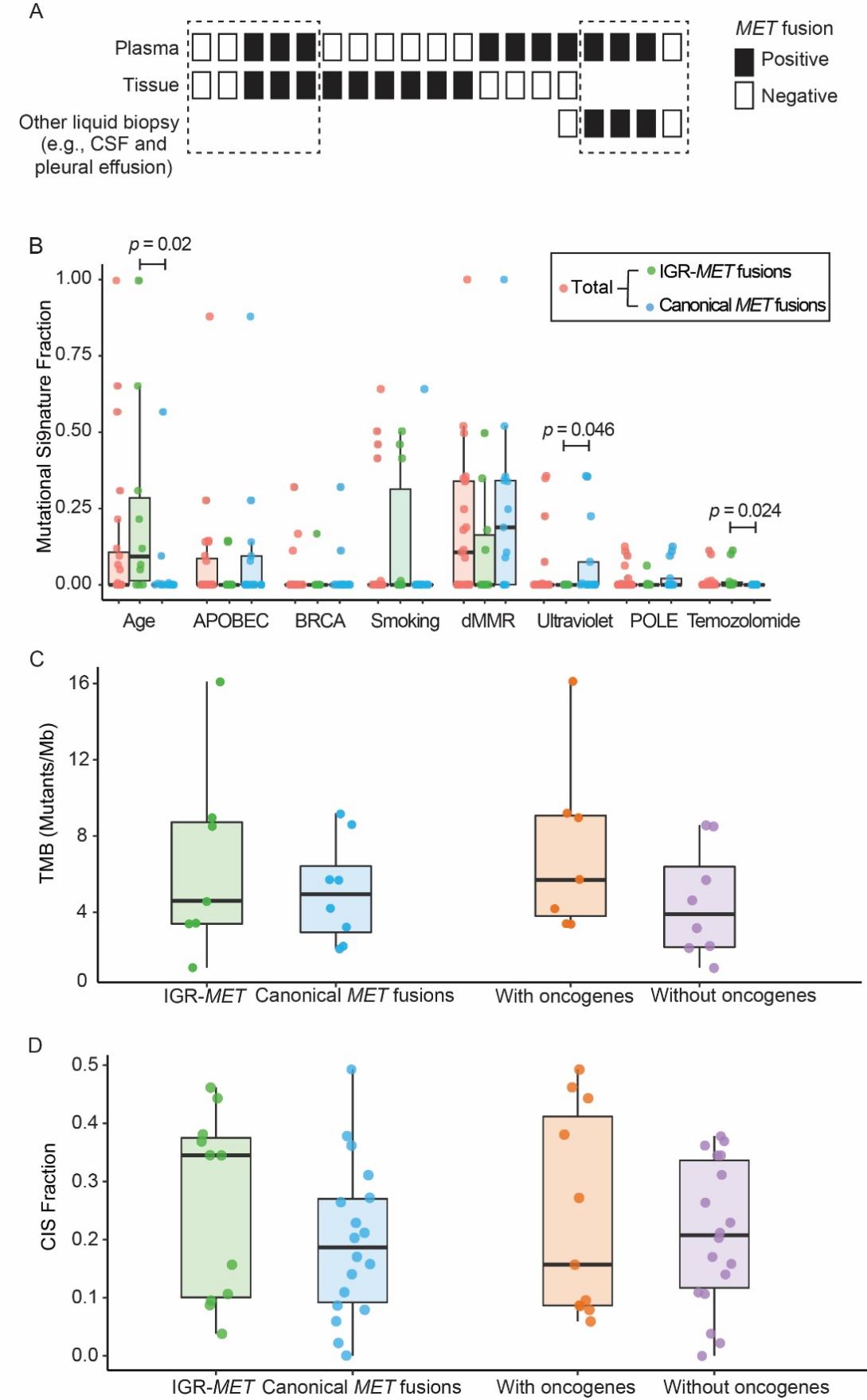


**Supplementary Figure S1. Concordance of *MET* fusion detection in multiple sample types and subgroup analysis of TMB and CIS in baseline samples**

(A) The status of *MET* fusion detection (positive vs. negative) in 19 patients with two or more baseline samples are shown. The dashed squares highlight the samples with concordant *MET* fusion detection. (B) The mutational signature analysis is performed in 425-gene NGS panel-sequenced baseline samples, which is further compared between IGR-*MET* and canonical *MET* fusion subgroups. (C-D) TMB and CIS levels are compared between subgroups of IGR-*MET* and canonical *MET* fusions as well as subgroups with and without concurrent oncogenes including *EGFR*, *ALK*, *KRAS*, *RET*, *HER2*, *BRAF*, *NTRK1/2/3,* and *ROS1*. Only the tissue samples sequenced using the 425-gene NGS panel were included for TMB analysis. Statistical analyses showed no significant differences in all comparisons.

Abbreviations: CSF, cerebrospinal fluid; TMB, tumor mutational burden; CIS: chromosome instability.
